# Pharmacological Characterisation of Nicotinic Acetylcholine Receptors Expressed in Human iPSC-Derived Neurons

**DOI:** 10.1371/journal.pone.0125116

**Published:** 2015-04-23

**Authors:** Anna Chatzidaki, Antoine Fouillet, Jingling Li, Jeffrey Dage, Neil S. Millar, Emanuele Sher, Daniel Ursu

**Affiliations:** 1 Department of Neuroscience, Physiology & Pharmacology, University College London, London, United Kingdom; 2 Lilly Research Centre, Eli Lilly and Company, Windlesham, Surrey, United Kingdom; 3 Lilly Research Laboratories, Eli Lilly and Company, Indianapolis, Indiana 46285, United States of America; University of Exeter, UNITED KINGDOM

## Abstract

Neurons derived from human induced pluripotent stem cells (iPSCs) represent a potentially valuable tool for the characterisation of neuronal receptors and ion channels. Previous studies on iPSC-derived neuronal cells have reported the functional characterisation of a variety of receptors and ion channels, including glutamate receptors, γ-aminobutyric acid (GABA) receptors and several voltage-gated ion channels. In the present study we have examined the expression and functional properties of nicotinic acetylcholine receptors (nAChRs) in human iPSC-derived neurons. Gene expression analysis indicated the presence of transcripts encoding several nAChR subunits, with highest levels detected for α3-α7, β1, β2 and β4 subunits (encoded by *CHRNA3-CHRNA7*, *CHRNB1*, *CHRNB2* and *CHRNB4* genes). In addition, similarly high transcript levels were detected for the truncated dupα7 subunit transcript, encoded by the partially duplicated gene *CHRFAM7A*, which has been associated with psychiatric disorders such as schizophrenia. The functional properties of these nAChRs have been examined by calcium fluorescence and by patch-clamp recordings. The data obtained suggest that the majority of functional nAChRs expressed in these cells have pharmacological properties typical of α7 receptors. Large responses were induced by a selective α7 agonist (compound B), in the presence of the α7-selective positive allosteric modulator (PAM) PNU-120596, which were blocked by the α7-selective antagonist methyllycaconitine (MLA). In addition, a small proportion of the neurons express nAChRs with properties typical of heteromeric (non-α7 containing) nAChR subtypes. These cells therefore represent a great tool to advance our understanding of the properties of native human nAChRs, α7 in particular.

## Introduction

Nicotinic acetylcholine receptors (nAChRs) represent a diverse family of neurotransmitter-gated ion channels that are expressed at the neuromuscular junction (muscle-type nAChRs), in the nervous system (neuronal nAChRs), as well as in several peripheral tissues. Sixteen different nAChR subunits have been identified in humans (α1-α7, α9, α10, β1-β4, γ, δ and ε) that can assemble as pentamers to form a large number of different receptor combinations [1]. Most nAChRs contain more than one type of subunit (heteromeric nAChRs) whereas some subunits, such as α7, are capable of forming homomeric receptors, containing five copies of a single subunit. In addition to the gene encoding the nAChR α7 subunit (*CHRNA7*) a partially duplicated variant (*CHRFAM7A*) has been identified in the human genome [2,3] and both of these genes (*CHRNA7* and *CHRFAM7A*) have been linked to schizophrenia [4–7]. *CHRFAM7A* encodes a fusion protein (dupα7), corresponding to the ion channel domain of α7 fused to an unrelated gene at its N-terminus There is evidence, admittedly only in recombinant systems, that dupα7 can co-assemble with the α7 subunit, and exert a dominant-negative effect, resulting in reduced functional expression of α7 nAChRs [8,9].

Neuronal nAChRs have been implicated in a variety of neurological and psychiatric disorders, including Alzheimer’s disease, schizophrenia, depression and attention deficit- hyperactivity disorder [10–13]. As a consequence, there has been a considerable interest, from both academic laboratories and pharmaceutical companies, in developing novel subtype-selective nAChR ligands [14,15]. For this reason, the identification of novel cellular assays, in particular those providing access to native human nAChRs, is an important discovery goal.

Induced pluripotent stem cells (iPSCs) can be generated from somatic cells by retroviral expression of reprogramming factors [16] and, by using a combination of growth factors and culture conditions, iPSCs can be further differentiated into a large variety of cellular types, including CNS-like neurons and glial cells [17,18]. Although it is possible to study the pharmacological properties of receptors expressed in naturally occurring neuronal preparations (for example, those obtained from neuronal tissues ablations), iPSC-derived neurons provide a more readily available source and have the potential to increase our understanding of how native human receptors function. In addition, iPSC-derived neurons can be generated from patients carrying a specific genetic background, corresponding to a particular neuropsychiatric disease. Indeed, recent studies using iPSC-derived neurons generated from patients have been successful in demonstrating phenotypes associated with a variety of diseases, such as Phelan—McDermid, Timothy, and Rett syndromes [19–25].

Many of the initial studies on human iPSC-derived neurons focused on the assessment of the expression of specific neuronal markers [17,18]. However, subsequent studies have demonstrated the functional expression of a variety of ion channels and neurotransmitter receptors [26,27]. The human iPSC-derived neurons used in the this study have been characterised previously by our group, both in terms of their general gene expression profile and the functional expression of various ion channels [27]. In addition, other groups have examined the electrophysiology profile of these cells [26]. The microarray data reported in [27] points to a gene expression profile that closely resembled that observed in neonatal prefrontal cortex tissue. In particular, genes which are known to be associated with neurite outgrowth, synaptic development or neuronal function were clearly expressed and upregulated in the iPSC-derived neurons [27]. In the present study, we have examined, by quantitative PCR, the profile of nAChR gene expression in the same line of human iPSC-derived neurons. Most importantly we have characterised the functional properties of nAChRs expressed in the iPSC-derived neurons by means of calcium flux assays, performed at either the single cell level or by a higher-throughput 96-well assay. In addition, we have confirmed that functional nAChRs are expressed in these cells by use of the patch-clamp electrophysiological technique.

## Materials and Methods

### Materials

(R)-N-(1-azabicyclo[2.2.2]oct-3-yl)(5-(2-pyridyl)thiophene-2-carboxamide) (compound B) was synthesised by Lilly Research Laboratories according to methods described previously [28]. 4-(1-napthyl)-3*a*,4,5,9*b*-tetrahydro-3*H*-cyclopenta[*c*]quinoline-8-sulfonamide (TQS) and 4-(4-bromophenyl)-3*a*,4,5,9*b*-tetrahydro-3*H*-cyclopenta[*c*]quinoline-8-sulfonamide (4BP-TQS) were obtained from Chembridge Corporation (San Diego, CA). PNU-120596, NS-1738 and 5-Iodo-A-85830 were obtained from Tocris Biosciences (Bristol, UK). 4-(5-(4-Chlorophenyl)-2-methyl-3-proprionyl-1*H*-pyrrol-1-yl)-benzenesulfonamide (A-867744) was provided by Abbott laboratories. Human neurons (iCell-Neurons) derived from induced pluripotent stem cells were obtained from Cellular Dynamics International (Madison, WI, USA). As outlined in the product specifications and the corresponding user guide, the iCell-Neurons were generated by using the proprietary differentiation and purification protocols provided by the supplier (see product userguide at and http://www.cellulardynamics.com/products/lit/CDI_iCellNeuronsUsersGuide140826.pdf).

According to the information provided by the supplier (CDI) they represent a mixture of post-mitotic neural subtypes comprising primarily of GABAergic and glutamatergic neurons.

### Gene expression examined by quantitative PCR

Cells (iPSC-derived neurons) were plated onto 10 cm dishes at a density of 2 x 10^6^ cells per dish, according to the manufacturer’s protocol. Three days after plating, total RNA was extracted using RNeasy Mini kit from Qiagen (Valencia, CA) according to the manufacturer’s instruction. On-Column DNase Digestion with the RNase-Free DNase Set (Qiagen, Valencia, CA) was performed to remove residual DNA contamination. Total RNA was quantified via a Nanodrop spectrophotometer ND-1000 (Thermo Scientific, Wilmington, DE) and stored at −80°C. First strand cDNA synthesis was performed using High-Capacity cDNA Reverse Transcription kit from Life Technologies (Foster City, CA) according to the manufacturer’s protocol using a thermo cycler (Thermo Scientific) at 25°C for 10 minutes, 37°C for 120 minutes, and 85°C for 5 minutes. Gene specific TaqMan gene expression assays were purchased from Life Technologies (Foster City, CA). PCR reactions of 50 ng, 10 ng, 2 ng, 0.4 ng and 0.08 ng RNA input per reaction were carried out with TaqMan Universal PCR Master Mix (Life Technologies) in triplicate for each gene and run on an ABI 7900HT Fast Real-Time PCR system (Life Technologies) under the following conditions: 60°C for 2 minutes, 95°C for 10 minutes, 95°C for 15 sec for 40 cycles, 60°C for 1 minute. Transcript levels of TATA-box binding protein (TBP) were measured as endogenous controls. Gene expression was analyzed based on the delta CT approach and normalized to the expression of TBP. The specificity of the PCR products was assessed by analysis of the 50 ng PCR products by gel electrophoresis on a 2% agarose gel and comparison with a 100 bp DNA ladder.

### FLIPR-based intracellular calcium assay

Frozen aliquots of iPSC-derived neurons were thawed and plated at a density of 2.5 x 10^5^ cells/ml (50 μl per well) on poly(D)-lysine (PDL)/laminin-coated black-walled clear bottom, half area 96-well plates (Corning). Cells were maintained in a humidified incubator containing 5% CO_2_ at 37°C. Cells were assayed in a fluorescent imaging plate reader (FLIPR, Molecular Devices Corporation, UK) 4–8 days after plating (unless otherwise specified). A modified HEPES-buffered Tyrode’s solution (HBTS) (Invitrogen, Paisley, UK) was used throughout the experiments, containing (mM): 135 NaCl, 5 KCl, 1.2 MgCl_2_, 2.5 CaCl_2_, 10 HEPES, 10 glucose pH 7.2). Cell medium was removed and the cells were incubated in 50 μl of 1 μM Fluo-4 acetoxymethyl ester (Invitrogen) in HBTS with 0.05% Pluronic F-127 (Invitrogen) for 60 min at room temperature, protected from light. Fluo-4 was then removed and 50 μl HBTS were added in each well. The cells were then assayed using a FLIPR. Cells were excited by light at 488 nm from a 4W Argon-ion laser and the emitted fluorescence passed through a 510–570 nm band-pass interference filter before detection with a cooled charge coupled device (CCD) camera (Princeton Instruments). Drug dilutions in assay buffer were prepared in a separate 96-well, flat-bottom plate. Parameters for drug addition to the cell plate were pre-programmed, and delivery was automated through a 96-well head pipettor. Drugs were added in 25 μl volumes by automated pipetting. Intracellular calcium levels were monitored before and after the addition of the compounds. Fluorescence data were exported and analysed in Microsoft Excel and GraphPad Prism. Data are presented as normalized values, where the baseline fluorescence was subtracted from the peak fluorescence and then expressed as a percentage of the response obtained by depolarising the cells with 30 mM KCl. All data presented in concentration-response curve format correspond to averages ± SEM of 3–5 independent experiments performed on different days by using different batches of cells. For each single experiment the data were calculated by averaging a minimum of 3 replicates (i.e. wells). Concentration—response curves were fitted by a non-linear least-squares fit algorithm according to the equation: E = E_max_/(1+{EC_50_/[conc]^nH^) in which E_max_ is the maximum obtainable response, EC_50_ is the concentration of the agonist that elicits 50% of the maximum obtainable response, and n_H_ is the Hill slope. Results are expressed as mean ± SEM. Student's two-sided t test was used to test for significant differences of mean values (assuming two independent populations; P < 0.05).

### Single cell calcium imaging

iPSC-derived neurons were thawed, plated and cultured using the same protocol as specified above for the FLIPR experiments. Fluorescence-based calcium imaging experiments were carried out 6–10 days after plating. After loading the cells with the calcium sensitive dye (performed as for the FLIPR experiments) cells were washed and continuously perfused during the experiment with HBTS. The perfusion flow rate was 3 ml/min, which results in complete replacement of the 100 μl volume in each well every 2 seconds. Dye-loaded cells were viewed using an inverted epifluorescence microscope (Axiovert, 135TV, Zeiss, Cambridge, UK). Fluo-4 fluorescence was excited by a 480 ± 10 nm light source (Polychrome II, TILL-Photonics, Gräfelfing, Germany) and emission was captured by an iXon 897 EMCCD camera (Andor Technologies, Belfast, UK) after passage through a dichroic mirror (505LP nm) and a high pass barrier filter (515LP nm). Digitised images were stored and processed by using Imaging Workbench 5.0 software (INDEC Biosystems, Santa Clara, CA, USA). Data were analysed by averaging individual traces collected from a large number of cells in multiple wells of the 96-well plate. Delta F/F_0_ values were measured by quantifying the ratio between the change in fluorescence signal intensity (delta F) and baseline fluorescence (F_0_).

### Patch-clamp Recording

Frozen aliquots of iPSC-derived neurons were thawed and plated on Biocoat glass cover slips (BD Biosciences) at a density of 10,000 cells per coverslip and maintained in a humidified incubator containing 5% CO_2_ at 37°C. Whole-cell voltage-clamp recordings were carried out 6–10 days after plating. During recordings, cells were continuously perfused in HBTS at room temperature. Cells were voltage-clamped in the whole-cell configuration (at a holding potential of -60 mV) with an AxoPatch 200A patch-clamp amplifier (Molecular Devices, Sunnyvale, CA, USA). Pipettes were pulled from borosilicate glass (Type GC150TF-10, Harvard Apparatus, Kent, UK) using a commercial puller (Model P-87, Sutter Instruments, Novato, CA, USA) and had resistances between 2 and 6 MΩ when filled with a pipette solution containing (mM): 1 MgCl_2_, 4 MgATP, 0.5 EGTA, 10 HEPES, 140 K gluconate (pH adjusted to 7.3 with KOH). Currents were recorded at 10 kHz using a DA/AD interface (Digidata 1322A, Molecular Devices, Sunnyvale, CA, USA). Drugs were applied using a multichannel perfusion system (Model BPS-8, Scientifica, Uckfield, UK) positioned 150 μm away from the recorded cell and controlled by Clampex 9 software (Molecular Devices, Sunnyvale, CA, USA).

## Results

### Expression of nAChR subunits

**Fig 1:**
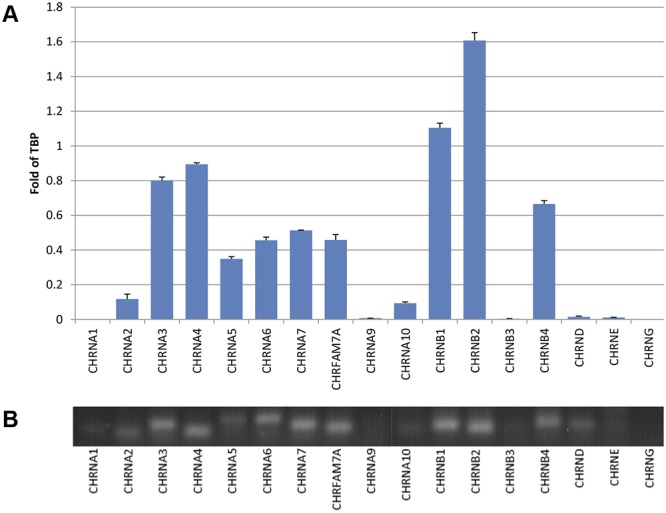
Relative Expression of nAChR subunits examined by RT-PCR. A) Levels of gene expression relative to TBP, using data generated from 10 ng RNA input. The following transcripts were either undetectable or detected only at very low levels at 10 ng input RNA: *CHRNA1* (encoding the α1 nAChR subunit), *CHRNA2* (α2), *CHRNA9* (α9), *CHRNA10* (α10), *CHRNB3* (β3), *CHRNG* (γ), CHRND (δ) and CHRNE (ε). Note, all of the CT values at lower input levels were near 35 or not detected. In contrast, the following transcripts were detected at relatively high levels: *CHRNA3-CHRNA7* (α3-α7), *CHRNB1* (β1), *CHRNB2* (β2), *CHRNB4* (β4) and the partially duplicated gene B) Agarose gel electrophoresis using PCR products from the 50 ng input.

Quantitative PCR was performed on first-strand cDNA that was prepared from iPSC-derived neurons. Using gene-specific primers, the abundance of mRNA for individual nAChR subunits was determined (Fig 1) using five levels of RNA input to ensure linearity and quality of the determination. The muscle-type nAChR subunit transcripts α1, γ, δ and ε subunits (encoded by the genes *CHRNA1*, *CHRNG*, *CHRND* and *CHRNE*, respectively) were either undetectable or detected at only very low levels (Fig 1). In addition, several neuronal nAChR subunit transcripts (α2, α9, α10 and β3 subunits; encoded by the *CHRNA2*, *CHRNA9 CHRNA10* and *CHRNB3* genes, respectively) were either not present or detected at only low levels. All other human nAChR subunit transcripts (α3-α7, β1, β2 and β4 subunits, encoded by the *CHRNA3*-*CHRNA7*, *CHRNB1*, *CHRNB2* and *CHRNB4* genes, respectively) were detected at broadly similar levels. In addition, transcripts of *CHRFAM7*, encoding dupα7, a partially duplicated variant of the α7 subunit gene *CHRNA7* [2,3] were also identified (Fig 1)

### Characterisation of nAChRs with a FLIPR-based assay

**Fig 2:**
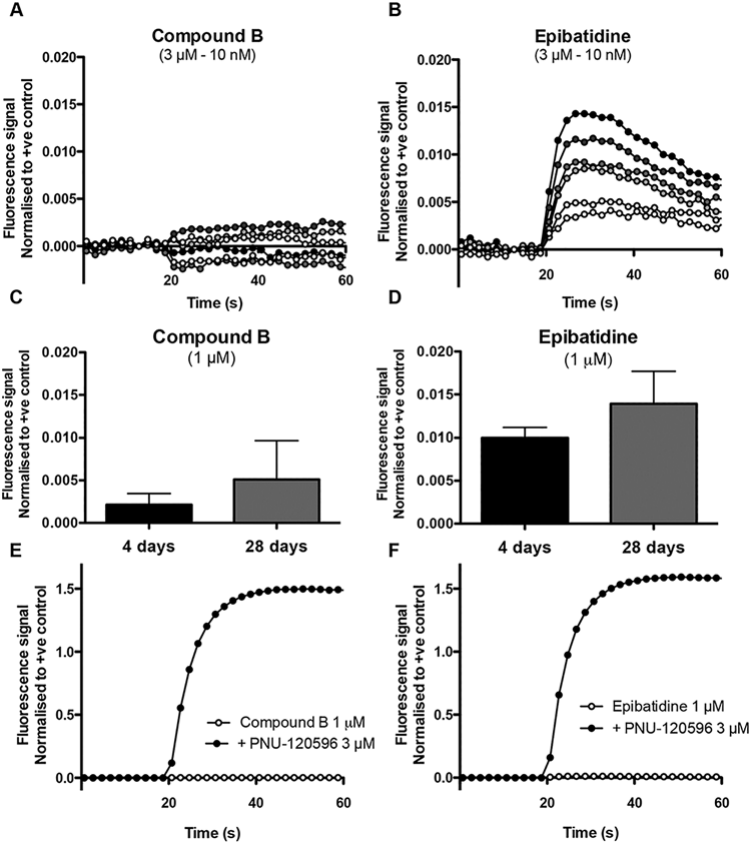
Nicotinic agonist-induced responses in iPSC-derived neurons examined by FLIPR. Representative examples of changes in fluorescence detected in iPSC-derived neurons with a range of concentrations of compound B (10 nM—3 μM; A) and epibatidine (10 nM—3 μM; B). C) and D) Averaged data for agonist induced responses in experiments performed at 4 and 28 days in culture. Data represent average of replicates ± SEM. Co-application of the α7-selective PAM PNU-120596 (3 μM; pre-applied for 60 s) with either compound B (1 μM; E) or epibatidine (1 μM; F) resulted in large fluorescence responses. Data are means ± SEMs from 3 experiments. All values are normalised to a control response to application of KCl (30 mM).

Based on the evidence obtained from quantitative PCR experiments, indicating that iPSC-derived neurons express a variety of nAChR subunit mRNAs, the expression of functional nAChRs was examined by a fluorescence-based calcium assay (Fig 2). Cells were loaded with a calcium-sensitive fluorescent dye (Fluo-4) and examined in a 96-well format fluorescent imaging plate reader (FLIPR). No agonist-induced intracellular calcium responses were detected with the α7-selective orthosteric agonist compound B (Fig 2A). This is in agreement with previous studies of α7 nAChRs examined by fluorescence-based methods [29] and is likely to be a consequence of the low open probability and fast desensitisation of α7 nAChRs that is observed in response to activation by orthosteric agonists [29,30]. In contrast to α7 nAChRs, which display very rapid desensitisation, most nAChRs desensitise relatively slowly during prolonged agonist applications and might be expected to be detected more easily in fluorescence assays using conventional orthosteric agonists. Application of epibatidine, a non-selective agonist of neuronal nAChRs, resulted in a detectable fluorescence response in iPSC-derived neurons (Fig 2B). However, although a response to epibatidine was detectable in these cells, it generated a relatively small signal, being only 1 ± 0.2% of a control response obtained by depolarisation of cells with KCl (Fig 2B; n = 3). Responses evoked by compound B (1 μM) and epibatidine (1 μM) were also studied at different culture times (4 days vs 28 days) (Fig 2C and 2D). The data obtained at longer time in culture failed to show a significant increase in response amplitude. For compound B, the response was 0.2 ± 0.1% of the positive control after 4 days in culture, while after 28 days in culture the response was 0.5 ± 0.5% of the positive control (P = 0.65, n = 3). For epibatidine, the response was 1.0 ± 0.1% of the positive control after 4 days in culture and 1.4 ± 0.4% of the positive control after 28 days in culture (P = 0.38, n = 3 replicates). In contrast to the small responses observed to nicotinic agonists applied alone, a strong increase in intracellular calcium signal was observed when either compound B or epibatidine were co-applied with the α7-selective positive allosteric modulator (PAM) PNU-120596 (Fig 2E and 2F). As reported previously, PNU-120596 dramatically reduces desensitisation kinetics of α7 nAChRs [29,31,32]. When co-applied with PNU-120596, large responses were observed with both compound B and epibatidine (119 ± 2% and 110 ± 5% of control KCl responses, respectively; n = 3–5).

### Characterisation of nAChRs by single cell calcium imaging

**Fig 3:**
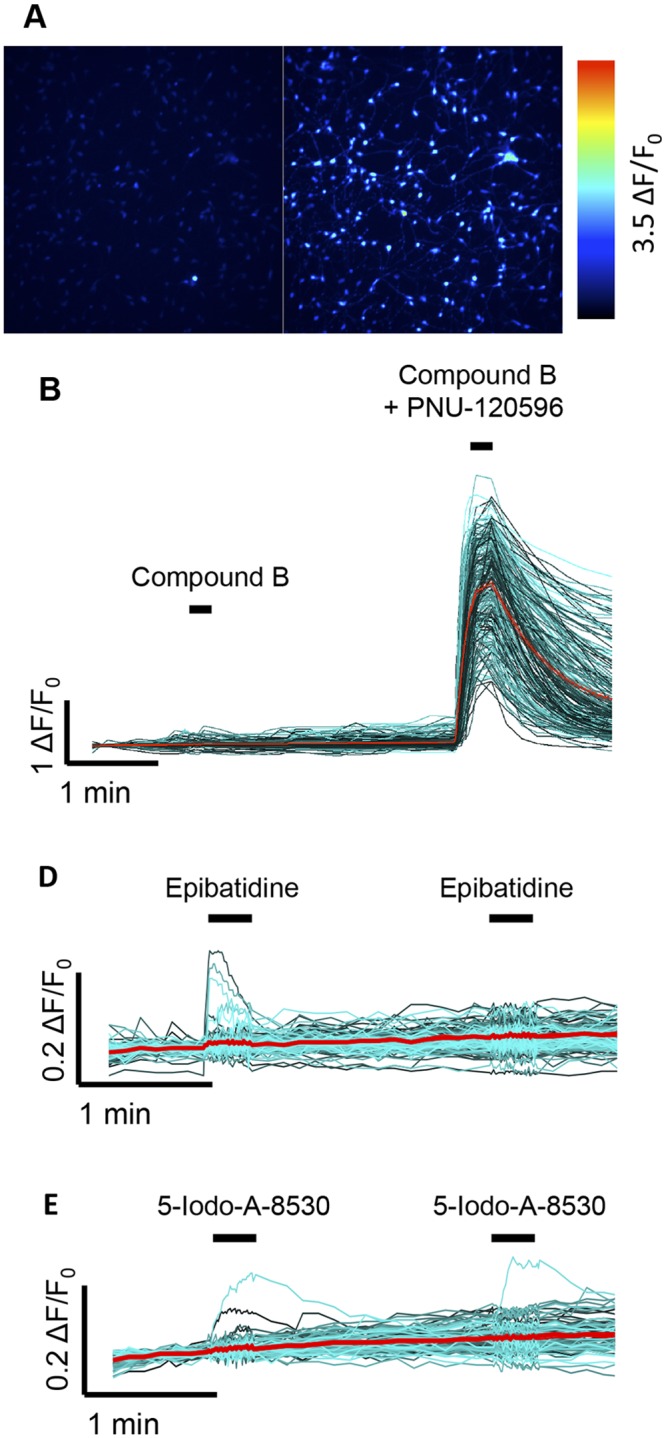
Characterisation of nAChRs in iPSC-derived neurons, examined by single-cell intracellular calcium imaging. A) Pseudocolour images of human iPSC-derived neurons corresponding to low initial resting calcium levels (Left panel) and higher calcium levels after co-application of compound B (1 μM) with PNU-120596 (3 μM) (Right panel). B) Single-cell traces for neurons present in the optical field, showing the effects of application of compound B (1 μM) followed by co-application of compound B (1 μM) with PNU-120596 (3 μM). C) Single-cell traces in response to two consecutive applications of epibatidine (1 μM). D) Single-cell traces in response to two consecutive applications of 5-Iodo-A-85380 (1 μM). In B-D, individual single-cell traces are displayed in cyan, whereas a mean response (average of multiple cells) is shown in red.

In order to obtain information about the heterogeneity of nicotinic responses in iPSC-derived neurons at the single-cell level, cell monolayers were examined by fluorescence-based intracellular calcium imaging (Fig 3). In agreement with FLIPR-based measurements (Fig 2), no response was detected to compound B when applied alone, whereas a large change in fluorescence was observed when compound B was co-applied with PNU-120596 (Fig 3A and 3B). In contrast to the absence of response to compound B, a small proportion of cells (4.9%) responded to application of epibatidine (Fig 3C). As illustrated in a representative example (Fig 3C), the second application of epibatidine did not cause an increase in calcium in the cells that had responded after the first application, presumably because of residual desensitisation to the initial agonist application. Similarly, only a very small number of cells (3.7%) responded to the α4β2-selective agonist 5-Iodo-A-85380 (Fig 3D), suggesting that there are only a small population of cells expressing functional α4β2 receptors.

### Effect of temperature on agonist-induced nAChR responses

**Fig 4:**
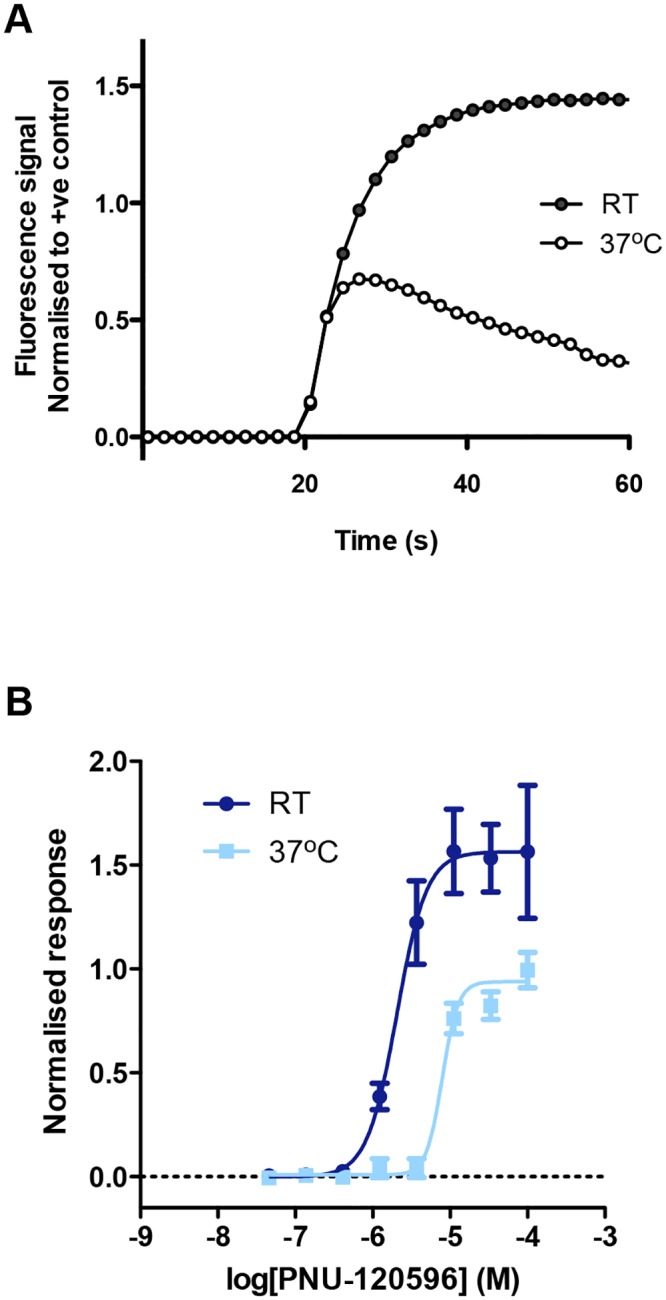
Influence of temperature on potentiation of Compound B responses by PNU-120596. A) Representative FLIPR traces showing the change in fluorescence observed when compound B (1 μM) and PNU-120596 (100 μM) were co-applied to iPSC-derived neurons at room temperature (RT) (closed circles) and at 37°C (open circles). B) Concentration-response relationship of PNU-120596 in the presence of compound B (1 μM) at room temperature (RT) and at 37°C. Data points are means of 4 independent experiments each of which generated paired data from the same batch of cells incubated at two temperatures.

The FLIPR and single-cell imaging experiments described previously were all performed with cells maintained at room temperature during agonist application. However, previous studies with recombinant nAChRs expressed in *Xenopus laevis* oocytes have indicated that the magnitude of nAChR responses can be influenced by temperature [33–35]. When such experiments are performed at physiological temperature (37°C), rather than at room temperature, increased responses have been observed for α4β2 nAChRs [34] and decreased responses for α7 nAChRs [33–35]. For this reason, FLIPR assays with iPSC-derived neurons were compared at room temperature and at 37°C. No significant differences were observed in responses to either compound B or epibatidine when applied alone. However, responses of reduced magnitude and different kinetics were observed at 37°C when compound B was co-applied with PNU-120596 (Fig 4). The maximum potentiated response at 37°C was significantly lower than that at room temperature (63.5 ± 5.4%, P = 0.04, n = 4) (Fig 4B), in agreement with previous studies [33–35]. Because of the relatively low proportion of cells expressing functional non-α7 receptors and our inability to enhance levels of functional expression in studies conducted at higher temperature, our subsequent studies were focused on understating in greater detail the pharmacological properties of the nAChRs expressed in these neurons that were activated by the α7-selective agonist compound B.

### Characterisation of α7 nAChRs expressed in iPSC-derived neurons

**Fig 5:**
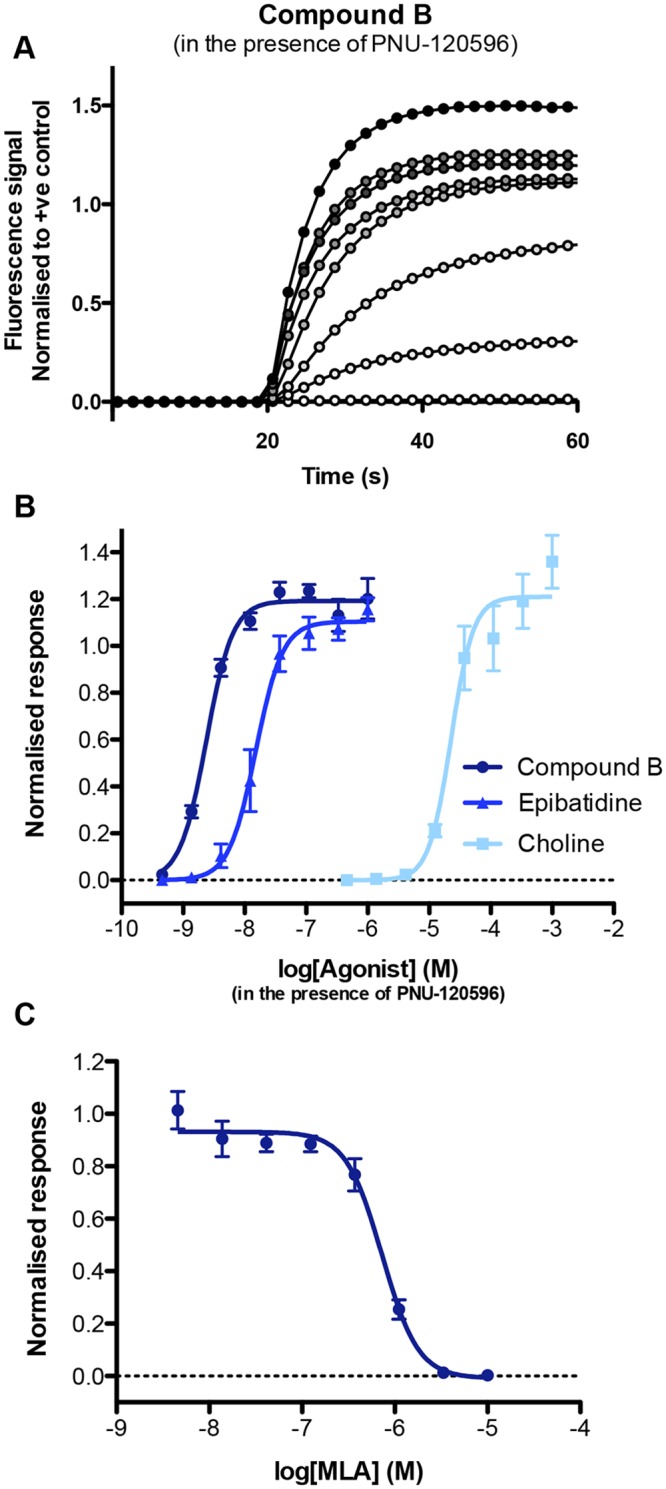
Potentiation and antagonism of nAChR agonist responses in iPSC neurons. (A) Representative of FLIPR traces produced with a range of compound B concentrations (0.3 nM—1 μM) in the presence of PNU-120596 (3 μM). Also shown are concentration-response curves for the agonists compound B (circles), epibatidine (triangles) and choline (squares), in the presence of PNU-120596 (3 μM) (B). Responses to compound B (1 μM) in the presence of PNU-120596 (3 μM) were blocked completely in a concentration-dependent manner by the α7-selective antagonist MLA (C). Data are means ± SEM of 3–5 independent experiments.

The data described previously (Figs 2–4) are consistent with the conclusion that α7 nAChRs are the major functional nicotinic receptor subtype expressed in iPSC-derived neurons. Further studies were performed to examine in more detail the pharmacological properties of nAChRs in these cells. As discussed earlier, α7 nAChRs are characterised by very fast desensitisation kinetics and, therefore, agonist responses alone are not easily detected using calcium imaging. For this reason, responses to a range of concentrations of three agonists (compound B, epibatidine and choline) were examined in the presence of a fixed concentration of the α7-selective PAM PNU-120596. Representative FLIPR traces and concentration-response relationships are illustrated in Fig 5 and the mean ± SEM of the EC_50_, E_max_ and n_H_ values for 3–5 independent experiments are summarized in Table 1. Further evidence that these agonist-evoked responses in iPSC-derived neurons are due to activation of α7 nAChRs was provided by the α7-selective antagonist methyllcaconitine (MLA). Responses to compound B in the presence of PNU-120596 were blocked completely and in a concentration-depended manner by MLA (Fig 5C) with an IC_50_ value of 0.7 ± 0.1 μM (n = 3). This is very similar to the IC_50_ value for MLA (0.8 ± 0.1 μM) that has been determined previously for recombinant α7 nAChRs in the presence of PNU-120596 [36].

**Table 1:** Pharmacological properties of nAChR ligands on iPSC-derived neurons.

Compound	EC_50_	n_H_	Normalised E_max_
Epibatidine	0.38 ± 0.27 μM	1.8 ± 2.1	0.01 ± 0.002
Epibatidine (+ 3 μM PNU-120596)	14.2 ± 5.6 nM	2.0 ± 0.4	1.10 ± 0.05
Choline (+ 3 μM PNU-120596)	26.1 ± 3.2 μM	2.4 ± 0.5	1.21 ± 0.05
Compound B (+ 3 μM PNU-120596)	2.6 ± 0.3 nM	2.0 ± 0.2	1.19 ± 0.02
Compound B (+ 10 μM A-867744)	13.7 ± 3.1 nM	3.1 ± 1.3	0.79 ± 0.02
Compound B (+ 10 μM TQS)	1.6 ± 0.3 nM	2.3 ± 0.7	0.23 ± 0.01
Compound B (+ 30 μM NS-1738)	10.2 ± 5.0 nM	2.6 ± 1.6	0.04 ± 0.003
PNU-120596 (+ 1 μM Compound B)	1.5 ± 0.2 μM	1.7 ± 0.3	1.31 ± 0.03
A-867744 (+ 1 μM Compound B)	0.8 ± 0.1 μM	1.9 ± 0.9	1.81 ± 0.14
TQS (+1 μM Compound B)	0.6 ± 0.1 μM	2.2 ± 1.2	0.30 ± 0.02
NS-1738 (+ 1 μM Compound B)	2.3 ± 0.2 μM	2.8 ± 0.5	0.03 ± 0.001
4BP-TQS	4.3 ± 3.4 nM	2.2 ± 0.5	0.33 ± 0.06

Data are means ± SEM of 3–5 independent experiments.

**Fig 6:**
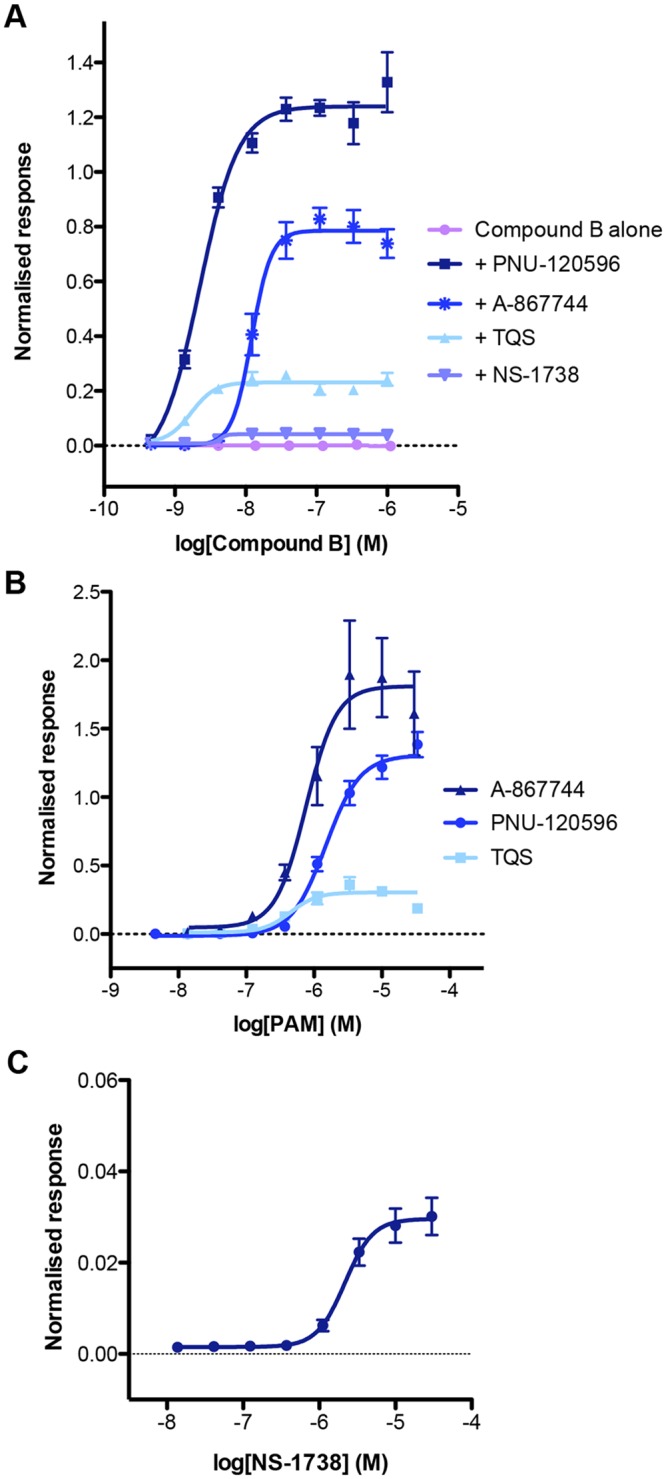
Characterisation of α7-selective type I and type II PAMs in iPSC-derived neurons. (A) Concentration-response relationship of Compound B in the presence of PNU-120596 (3 μM; squares), A-867744 (10 μM; asterisks), TQS (10 μM; triangles), NS-1738 (30 μM; inverted triangles) and in the absence of a PAM (circles). B) Concentration-response curves of the type II PAMs A-867744 (triangles), PNU-120596 (circles) and TQS (squares), in the presence of maximum concentration of Compound B (1 μM). C) Concentration-response relationship of the type I PAM NS-1738 in the presence of Compound B (1 μM). Data are means ± SEM of 3–5 independent experiments.

Additional FLIPR experiments were performed to compare a series of α7-selective PAMs, including those classified as type I PAMs (compounds that have no significant effect on agonist-induced desensitisation of α7 nAChRs) or as type II PAMs (those that reduce desensitisation). Concentration-response curves were constructed (Fig 6A) using a range of concentrations of compound B in the presence of either a fixed concentration of a type I PAM (NS-1738) or in the presence of one of three different type II PAMs (PNU-120596, A-867744 and TQS). As illustrated in Fig 6A, significantly larger increases in fluorescence were observed in the presence of type II PAMs (PNU-120596, A-867744 and TQS) than with the type I PAM NS-1738 (Table 1). A similar series of FLIPR experiments were performed in which a range of concentrations of the four α7-selective PAMs was examined in the presence of a fixed concentration of compound B (Fig 6B and 6C). As found previously, significantly larger maximal responses were observed with type II PAMs than with the type I PAM (Fig 6; Table 1).

**Fig 7:**
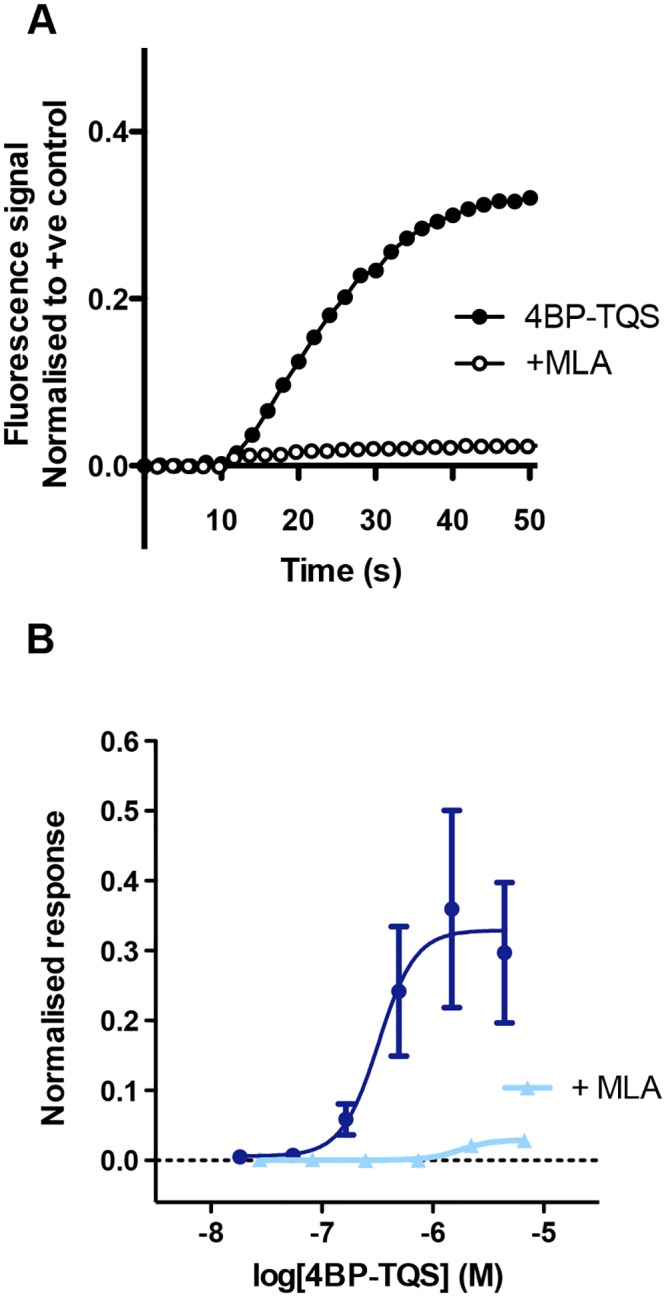
Agonist activity of 4BP-TQS in iPSC-derived neurons examined by FLIPR. (A) Representative examples of FLIPR traces with the α7-selective allosteric agonist 4BP-TQS (10 μM; closed circles). Responses to 4BP-TQS are inhibited by the α7-selective antagonist MLA (1 μM; open circles). (B) Concentration-response curve for 4BP-TQS CRC (circles) and 4BP-TQS in the presence of the α7-selective antagonist MLA (1 μM; triangles). Data are means ± SEM of 3 independent experiments.

In contrast to orthosteric agonists, such as acetylcholine, which induce rapid desensitisation of α7 nAChRs, 4BP-TQS is an example of an α7-selective allosteric agonist that has been reported to interact with a transmembrane binding site and cause receptor activation associated with minimal desensitisation [29,37]. In contrast to α7-selective orthosteric agonists (such as compound B) applied alone, clear concentration-dependent agonist responses were observed with 4BP-TQS (Fig 7; Table 1). The EC_50_ value for activation by 4BP-TQS was 4.3 ± 3.4 μM and responses to 4BP-TQS were blocked by MLA (Fig 7). Furthermore, block by MLA was not surmountable (Fig 7B), which is consistent with evidence that the 4BP-TQS and MLA bind non-competitively at distinct allosteric and orthosteric binding sites [37].

### Potentiated nAChR responses detected by patch-clamp recording

**Fig 8:**
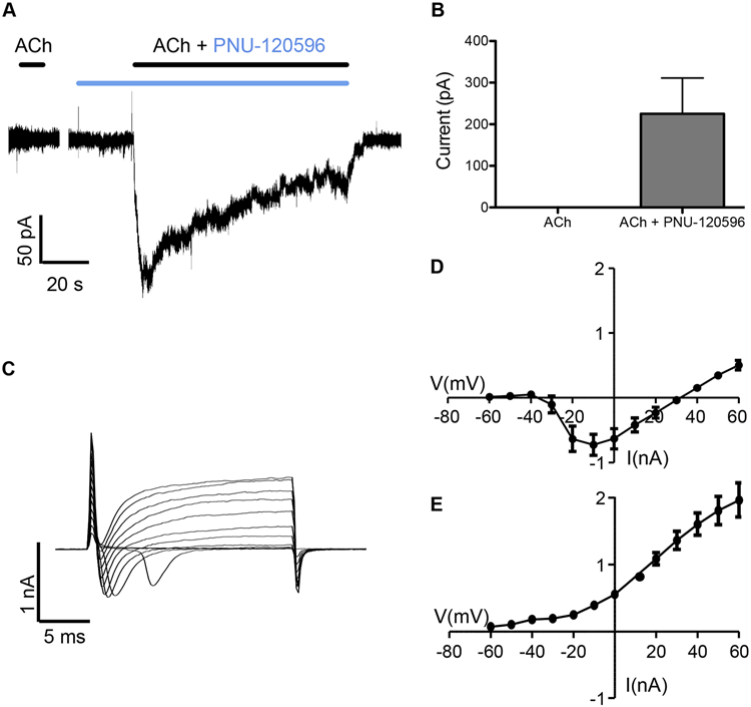
Agonist and voltage induced responses in iPSC-derived neurons examined by patch-clamp electrophysiology. A)Representative recording showing no detectable currents with acetylcholine (1 mM) (Left) and a slowly desensitising current in response to co-application of acetylcholine (1 mM; black bar) and PNU-120596 (3 μM; blue bar) (Right). PNU-120596 was pre-applied for 20 s before co-application with acetylcholine. B) The average response data from n = 5 cells was 225.2 +/- 85.9 pA. Representative voltage step current responses with Nav and Kv currents are shown in C). The fast component of the inward current was analyzed and plotted for the Na^+^ current (first 5 ms) and (n = 5 cells) in D. The steady-state current (last 5 ms) corresponding to currents through K^+^ channels is shown in E (n = 5 cells).

The ability of nicotinic agonists to activate nAChRs expressed in iPSC-derived neurons was also examined by patch-clamp recordings. Surprisingly, no agonist-evoked currents could be detected when acetylcholine (the endogenous agonist of nAChRs) was applied alone. However, in the experiments where acetylcholine was co-applied with PNU-120596, a large inward current was observed with a value of 225.2 ± 85.9 pA (n = 5). Fig 8A shows a representative patch-clamp trace from iPSC-derived neurons, illustrating the absence of a response to acetylcholine and a slowly desensitising current in response to the co-application of acetylcholine with PNU-120596. The pooled data from 5 different recorded cells are presented in Fig 8B. Under the same experimental conditions we have also recorded sodium and potassium currents induced by voltage steps. Fig 8C shows representative current traces obtained by subsequent application of increasing voltage steps ranging in amplitude from -60 to +60 mV. Fig 8D and 8E show characteristic current-voltage relationships for the inward Na^+^ current (analysed in the first 5 ms part of the step depolarisation) and slow outward K^+^ current (analysed at the last 5 ms of the voltage step).

## Discussion

iPSC-derived neurons provide a readily available supply of human cells with which to study endogenous neuronal nAChRs and also provide several opportunities for both pharmaceutical drug-discovery and academic research. Here, we have extended our previous studies of iPSC-derived neurons [27,29] with a detailed pharmacological characterization of nAChR subtypes expressed in these cells.

Quantitative PCR experiments indicated that the iPSC-derived neurons examined in this study express mRNA for a variety of nAChR subunits. However, despite this finding, functional characterisation (performed by FLIPR, calcium imaging and patch-clamp recording), suggests that α7 nAChRs are the predominant subtype of functional nicotinic receptor in these cells.

Initially, FLIPR assays were used to investigate the composition of the nAChR population expressed in these neurons. Application of the α7-selective agonist, compound B, did not elicit any detectable fluorescence responses. However, large responses were observed when compound B was co-applied with PNU-120596, an α7-selective PAM that reduces agonist-evoked desensitisation of α7 nAChRs. As has been discussed previously [29] difficulties in detecting responses to α7-selective agonists such as compound B using fluorescence-based assays are likely to be due to the very fast desensitisation of α7 nAChRs. In contrast, small, concentration-dependent responses were observed with epibatidine, a non-selective nicotinic agonist, which are likely to be due to the activation of heteromeric non-α7 nAChRs that desensitise more slowly.

Single-cell calcium imaging was used with the aim of examining the proportion of iPSC-derived neurons responding to nicotinic agonists. As was found with the FLIPR-based assay, α7 responses could only be detected when orthosteric agonists were co-applied with a PAM. While almost all cells in the optical field showed a large increase in intracellular calcium when compound B was co-applied with PNU-120596 (assumed to be cells expressing functional α7 nAChRs), fluorescence responses were detected in only a very small number of cells when the non-selective agonist epibatidine was applied alone. In addition, the α4β2-selective agonist, 5-Iodo-A-85380, gave similar results to epibatidine, suggesting that only a small number of cells express functional α4β2 nAChRs.

Previous studies have indicated that changes in temperature can influence the magnitude of agonist responses with neuronal nAChRs [33–35]. Experiments conducted with recombinant nAChRs have indicated an increase in responses with α4β2 nAChRs and a decrease in responses with α7 nAChRs when performed at physiological temperature (37°C), rather than at room temperature [33–35]. For this reason, functional responses in iPSC-derived neurons were examined at both room temperature (22°C; the standard experimental conditions used for the experiments reported in this study) and also at physiological temperature (37°C). No significant difference was observed when agonists such as compound B or epibatidine were applied alone but responses to agonists in the presences of α7-selective PAMs were lower at 37°C. This is consistent with heteromeric nAChRs such as α4β2 being a minor component in these cells and α7 nAChRs being the predominant nAChR subtype in iPSC-derived neurons

Perhaps not unexpectedly, the mRNA expression profile determined in the quantitative PCR study is not in direct agreement with the functional data. Although the expression profile suggests that mRNA for many neuronal nAChR subtypes is expressed by these cells, the majority of functional nAChRs detected in these studies have pharmacological properties that are characteristic of the α7 receptor subtype. It appears therefore that the expression profile of nAChR subunit mRNAs does not reflect the profile of functional nAChRs in these cells. A more detailed pharmacological characterisation, with a variety of agonists, antagonists and PAMs, was consistent with α7 receptors being the predominant functional nAChR subtype in iPSC-derived neurons.

Patch-clamp recordings were performed with the aim of investigating in more detail the properties of nAChRs expressed in iPSC-derived neurons. We have been unable to detect responses to a variety of nicotinic agonists (acetylcholine, epibatidine, choline or compound B) when applied alone. However, when these agonists were co-applied with PNU-120596, large, slow-desensitising inward currents were detected. This is consistent with previous evidence that α7 nAChRs have a low open probability and desensitise rapidly when activated by conventional orthosteric agonists, but have greater open probability and reduced desensitisation when orthosteric agonists are co-applied with type II PAMs [38–40]. The lack of agonist-induced α7 responses in the patch-clamp experiments can be also attributed to the general low expression of ion channels and receptors observed in the iPSC-derived neurons. Under identical experimental conditions, and with similarly fast drug applications, we were able to readily detect nicotinic currents in other cell types, such as rodent hippocampal neurons in culture [29].

In addition to transcripts for the α7 subunit (encoded by *CHRNA7*), our analysis of gene expression has provided evidence for expression in iPSC-derived neurons of the partially duplicated gene *CHRFAM7A*. Interestingly, both *CHRNA7* and *CHRFAM7A* have been implicated in cognitive disorders such as schizophrenia [6,7,41,42]. *CHRFAM7A* encodes a truncated version of the nAChR α7 subunit [3] which does not itself form a functional ion channel but it can reduce functional expression of α7 nAChRs via a dominant negative effect [8,9]. Our evidence for the expression of *CHRFAM7A* transcripts in iPSC-derived neurons indicates that these readily-available human neuronal cells may provide a valuable tool for studies aimed at investigating the role of *CHRFAM7A*. This is even more valuable considering that *CHRFAM7A* is only expressed in humans.

In summary, we have provided evidence that the predominant nAChR expressed as a functional receptor in iPSC-derived neurons has pharmacological properties typical of α7 nAChRs. These results have important implications for the development of drug-discovery focused screening assays on native receptors that could be used to identify new modulators of nAChRs in our quest to develop novel therapies for psychiatric and neurodegenerative disorders.
